# Seropositivity and associated intrinsic and extrinsic factors for Rift Valley fever virus occurrence in pastoral herds of Nigeria: a cross sectional survey

**DOI:** 10.1186/s12917-020-02455-8

**Published:** 2020-07-14

**Authors:** Nma Bida Alhaji, Jibrin Aminu, Mohammed Kabiru Lawan, Olutayo Olajide Babalobi, Ibrahim Ghali-Mohammed, Ismail Ayoade Odetokun

**Affiliations:** 1 Department of Public Health and Epidemiology, Niger State Ministry of Livestock and Fisheries, Minna, Nigeria; 2 Department of Veterinary Public Health and Preventive Medicine, Ahmadu Bello University, Zaria, Nigeria; 3 Department of Veterinary Public Health and Preventive Medicine, University of Ibadan, Ibadan, Nigeria; 4 Department of Veterinary Public Health and Preventive Medicine, University of Ilorin, Ilorin, Nigeria

**Keywords:** Cattle herds, Pastoralists, Rift Valley fever virus, Determinants, Sero-prevalence, Nigeria

## Abstract

**Background:**

Rift Valley fever (RVF) is a vector-borne emerging zoonotic disease of animals and humans, characterized by socioeconomic losses to livestock farmers and global public health threat. The study determined RVFV seroprevalence in cattle, assessed pastoralists’ knowledge about RVF, and factors that influence its occurrence in pastoral cattle herds of Nigeria. A cross-sectional study was conducted in pastoral herds of North-central Nigeria from 2017 to 2018. Data were collected using serology and questionnaire tools. Descriptive statistics were used to analyze the obtained data. Categorical variables were presented as proportions and their associations determined by Chi-square tests. Associations of risk factors were analyzed by univariable and multivariable logistic regressions analyses at 95% confidence level.

**Results:**

The overall IgM seropositivity of RVFV in pastoral cattle herds was 5.6%. This was higher in nomadic herds (7.4%) than in agro-pastoral herds (3.8%). All animal demographic characteristics of age, sex and breeds were not significantly (*p* > 0.05) associated with RVFV occurrence in pastoral herds. All the 403 pastoralists selected participated in the study, with the majorities of them being male, married and have no formal education. Majority of the pastoralists had low knowledge levels about zoonotic RVFV infection. All identified socio-ecological factors significantly (*p* < 0.05) influenced RVFV occurrence in herds. Mosquitoes availability in cattle environment (OR = 7.81; 95% CI: 4.85, 12.37), presence of rivers and streams at grazing fields (OR = 10.80; 95% CI: 6.77, 17.34), high rainfall (OR = 4.30; 95% CI: 2.74, 6.59), irrigated rice fields (OR = 5.14; 95% CI: 3.21, 7.79)**,** bushy vegetation (OR = 6.11; 95% CI: 3.96, 9.43), animal movement (OR = 2.2; 95% CI: 1.45, 3.25), and seasons (OR = 2.34; 95% CI: 1.55, 3.51) were more likely to influenced RVFV occurrence in cattle herds.

**Conclusions:**

Results of this study had illustrated recent circulation of RVFV in pastoral cattle herds in Nigeria and needs urgent interventions. The surveyed pastoralists had low knowledge level about RVF while the socio-ecological factors significantly influenced RVFV occurrence in herds. To address these gaps, pastoralists should be educated on clinical manifestations and modes of transmission of the disease in animals and humans, and mitigation measures. Adequate knowledge about RVF epidemiology will assure food security and public health.

## Background

Rift Valley fever (RVF) is a vector-borne emerging zoonotic disease of animals (cattle, small ruminants, camels, and wildlife) and humans, caused by RVF virus (RVFV) of the family *Bunyaviridae* and genus *Phlebovirus* [1, 2]. The disease causes significant morbidity and mortality of about 10 and 30%, respectively in animals [3]. Abortion is often the only obvious indication of the disease in cattle [4]. The virus is mainly transmitted among livestock through bites of mainly infected *Aedes* and *Culex* mosquitoes and possibly by bites of other infected blood-sucking insects, as well as by contacts with infected animal tissues, bodily fluids and fomites [1, 5, 6]. However, vertical transmission has also been reported [7]. RVFV is mostly transmitted to humans through bites of infected *Aedes* mosquitoes [8–10] and by direct contact with infected animals or inhalation of aerosols during the handling or slaughtering of infected ruminants [10, 11]. The disease causes major socioeconomic losses to livestock farmers and is a potential global public health threat [5, 12, 13].

RVF is endemic in many African countries, the Arabian Peninsula, and some Indian Ocean Islands [14, 15]. It is often encountered in endemic and epidemic forms in Africa and Middle East [3, 16, 17]. In West and Central Africa, its occurrence is associated with seasonal rainfall during non-epidemic periods [18]. For RVF occurrence, seasonal and ecologically driven risk factors are related to vector habitat availability and vegetation dynamics [19]. Movement of infected vectors, persons and animals could lead to emergence of the disease in non-endemic areas [20]. A clinical epizootic of RVF and its spread in the Sahel was associated with nomadic cattle and seasonal migrations of herdsmen [21].

Although no official report has indicated clinical RVF occurrence in Nigeria [22], studies have shown circulation of RVFV among ruminants and humans [23–25]. No attempt has been made to investigate current RVFV circulation and associated seasonal and socio-ecological influencing factors in the two major pastoral cattle production systems in Nigeria. Availability of such knowledge of the burden and exposure factors would facilitate the promotion of surveillance and control strategies for the virus. Effective surveillance and early warning systems for timely response to RVF emergence in the livestock population will require adequate knowledge about its epidemiology [26, 27]. The study objectives were: to determine seroprevalence of RVFV in nomadic and agro-pastoral cattle populations in North-central Nigeria; and assess pastoralists’ existing knowledge about RVF occurrence in herds. We hypothesized that intrinsic demographic characteristics of animals and extrinsic socio-ecological factors cannot influence emergence of RVFV in nomadic and agro-pastoral cattle herds in Nigeria.

## Results

### RVFV seropositivity

A total of 107 sera samples were screened for RVFV-IgM antibodies and six were seropositive for the virus. This finding represented animal-level anti-RVFV IgM antibodies recent burden of 5.61% (95% CI: 2.31–11.30) in pastoral herds of North-central Nigeria. A seroprevalence of 7.7% (95% CI: 2.00–19.52) was recorded in animals aged 1–3 years followed by those aged more than 3 years (4.4, 95% CI: 1.13–11.54). Under pastoral production systems, seroprevalence was higher in nomadic production system (7.4, 95% CI: 2.40–16.91) than in agro-pastoral system (3.8, 95% CI: 0.64–11.91). Details of breed, age, sex, and production system seropositivity are presented in Table 1.

**Table 1:** Anti-RVF IgM sero-prevalence in pastoral cattle herds of North-central Nigeria

Demographic characteristic	Number sampled	Number positive	Proportion (%)	95% CI
**Breed**				
Bokoloji	23	1	4.35	0.22, 19.63
Rahaji	26	1	3.86	0.19, 17.54
Bunaji	58	4	6.90	2.23, 15.80
**Age (in years)**				
1–3	39	3	7.70	2.00, 19.52
> 3	68	3	4.41	1.13, 11.54
**Sex**				
Bulls	24	2	8.33	1.42, 24.90
Cows	83	4	4.82	1.55, 11.21
**Production system**				
Agro-pastoral cattle	53	2	3.77	0.64, 11.91
Nomadic cattle	54	4	7.41	2.40, 16.91
**Total**	**107**	**6**	**5.61**	**2.31, 11.3**

*CI* Confidence interval

### Animal demographic characteristics associated with RVF occurrence

At univariable analysis, all animal demographic characteristics of age, sex, and breeds were not significantly (*p* > 0.05) associated with occurrence of RVF in pastoral cattle herds. No statistical association (χ2: 0.403, *p* = 0.810) was observed among the breeds. Among the age groups, there was no significant association in the seroprevalence (χ^2^:0.504, *p* = 0.470). Also, there was no significant association in seroprevalence between bulls and cows, as well as between agro-pastoral cattle and nomadic pastoral cattle [(χ^2^: 0.434, *p* = 0.510) and (χ^2^: 0.667, *p* = 0.410), respectively] as shown in Table 2.

**Table 2:** Animal demographic characteristics associated with occurrence of RVF virus in pastoral cattle herds of North-central Nigeria

Demographic characteristic	Number of samples negative n (%)	Number of samples positive n (%)	Chi-square (***X***^***2***^)	*P*-value
**Breed**				
Bokoloji	22	1	0.403	0.810
Rahaji	25	1		
Bunaji	54	4		
**Age (in years)**				
1–3	36	3	0.504	0.470
> 3	65	3		
**Sex**				
Bulls	22	2	0.434	0.510
Cows	79	4		
**Production system**				
Agro-pastoral cattle	51	2	0.667	0.410
Nomadic cattle	50	4		

Statistically significant at *p* < 0.05

### Demographic characteristics of participants

**Fig. 1:**
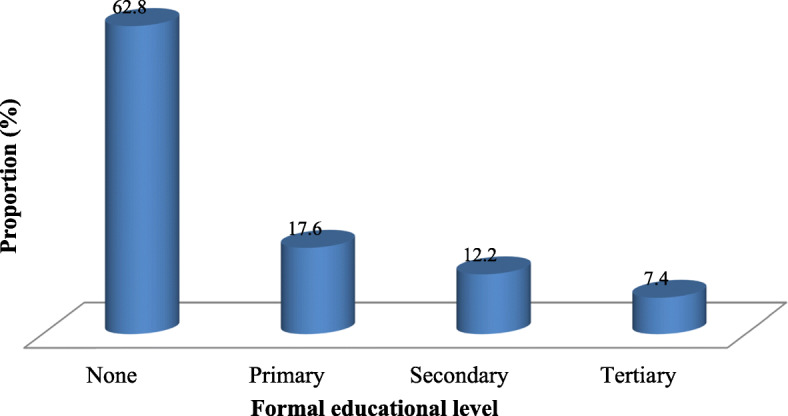
Pastoralists’ formal educational levels in pastoral settlements of Nigeria

All the 403 selected pastoralists, with mean age of 50.5 ± 15.5 years, participated in the study. Most of the participants (24.3%) were in the age group 50–59 years. The majority of respondents were male (81.9%; 95% CI: 77.9–85.42) and married (83.4%; 95% CI: 78.43–85.87), while 13.9% (95% CI: 10.77–17.54) and 3.7% (95% CI: 2.18–5.93) were single and widows, respectively. Based on occupation, 50.1% (95% CI: 45.25–55.00) of the participants were nomadic pastoralists and 49.9% (95% CI: 45.00–54.75) were agro-pastoralists. The majority of participants (62.8%; 95% CI: 57.98–67.40) had no formal education and very few (7.4%; 95% CI: 5.17–10.33) had tertiary education (Fig. 1).

### Knowledge about Rift Valley fever

All respondents reported having heard about RVF, locally called *Gabi-gabi*. Common sources of information about the disease were: relatives (93.4%) and radio (6.6%). When asked about clinical manifestations of RVF in cattle, majority of agro-pastoralists (67.79%) and nomadic pastoralists (92.6%) mentioned high mortality in newborns. Also, 85.1% agro-pastoralists and 94.6% nomadic respondents reported sudden onset of abortions in pregnant cows. However, few agro-pastoralists (27.9%) and nomadic (38.1%) mentioned high fever as sign in animals, and 43.8% agro-pastoralists and 82.7% nomadic indicated listlessness in newborns as clinical manifestation. Regarding mode of transmission of RVFV in cattle, most agro-pastoralists (57.7%) and nomadic (72.3%) reported mosquito bites, while 25.9% agro-pastoralists and 40.1% nomadic respondents mentioned bites of other flies. On zoonotic nature of RVF, very few agro-pastoralists (24.4%) and nomadic pastoralists (11.4%) mentioned the disease to be zoonotic. Most of the bivariate responses from two occupational groups on knowledge variables about the disease in cattle were significantly (*p* < 0.05) associated (Table 3).

**Table 3:** Knowledge about Rift Valley fever occurrence in pastoral settlements of North-central Nigeria

Variable	Pastoralists	No n (%)	Yes n (%)	***X***^***2***^	*P*-value
**Sign of RVF in cattle**					
High fever	Agro-pastoralist Nomadic	145 (72.1) 125 (61,9)	56 (27.9) 77 (38.1)	4.80	0.020
Anorexia	Agro-pastoralist Nomadic	72 (35.8) 13 (6.4)	129 (64.2) 189 (93.6)	52.87	< 0.001
High mortality in newborn calves	Agro-pastoralist Nomadic	65 (32.3) 15 (7.4)	136 (67.7) 187 (92.6)	39.3	< 0.001
Sudden onset of abortions	Agro-pastoralist Nomadic	30 (14.8) 11 (5.4)	171 (85.1) 191 (94.6)	9.8	0.001
Mucopurulent nasal discharge	Agro-pastoralist Nomadic	138 (68.7) 86 (42.6)	63 (41.3) 116 (57.4)	27.76	0.001
Listlessness in newborn calves	Agro-pastoralist Nomadic	113 (56.2) 35 (17.3)	88 (43.8) 167 (82.7)	65.58	< 0.001
Profuse fetid diarrhea	Agro-pastoralist Nomadic	163 (81.1) 117 (57.8)	38 (18.9) 85 (42.1)	25.51	0.001
**Mode of transmission of RVF in cattle**					
Bites of infected mosquitoes	Agro-pastoralist Nomadic	85 (42.3) 56 (27.7)	116 (57.7) 146 (72.3)	9.40	0.002
Bites of other biting flies	Agro-pastoralist Nomadic	149 (74.1) 121 (59.9)	52 (25.9) 81 (40.1)	9.23	0.002
Contacts with aborted foetus	Agro-pastoralist Nomadic	156 (77.6) 119 (58.9)	45 (22.4) 83 (41.1)	16.26	0.001
**Zoonotic nature of RVF**					
RVF can be transmitted from animals to humans	Agro-pastoralist Nomadic	152 (75.6) 179 (88.6)	49 (24.4) 23 (11.4)	11.59	0.001

*X*^*2*^ – Chi-square; Statistically significant at *p* < 0.05

### Socio-ecological drivers for Rift Valley fever occurrence in pastoral herds

At univariable analysis, all the socio-ecological predisposing factors and seasons were more likely to significantly (*p* < 0.05) influenced RVF occurrence in pastoral herds. However, multivariable logistic regressions revealed that: availability of mosquitoes in the pastoral environments was eight times more likely to influenced RVF occurrence (OR = 7.81; 95% CI: 4.85–12.37), while presence of rivers and streams in grazing fields was eleven times more likely to influenced RVF occurrence (OR = 10.80; 95% CI: 6.77–17.34). Also, high rainfall was more likely to influenced RVF occurrence in pastoral cattle herds (OR = 4.30; 95% CI: 2.74–6.59), and irrigated rice fields, and bushy vegetation were more likely to influenced emergence of the disease in herds [(OR = 5.14; 95% CI: 3.21–7.79) and (OR = 6.11; 95% CI: 3.96–9.43), respectively]. Furthermore, animal movement and seasons were twice more likely to influenced occurrence of RVF in pastoral cattle herds [(OR = 2.2; 95% CI: 1.45–3.25) and (OR = 2.34; 95% CI: 1.55–3.51), respectively] (Table 4).

**Table 4:** Socio-ecological factors that influence occurrence of RVF virus in pastoral cattle herds of North-central Nigeria

Factors	No influence n (%)	Yes influence n (%)	OR	95% CI	*P*-value
**High mosquitoes availability**					
Agro pastoralists	121 (60.2)	80 (39.8)	1.00		
Nomadic pastoralists	33 (6.1)	169 (93.9)	7.8	4.85, 12.37	< 0.001
**High cattle concentration**					
Agro pastoralists	110 (54.7)	91 (45.3)	1.00		
Nomadic pastoralists	63 (31.2)	139 (68.8)	2.7	1.78, 4.01	0.001
**High rainfall**					
Agro pastoralists	106 (52.7)	95 (47.3)	1.00		
Nomadic pastoralists	42 (20.8)	160 (79.2)	4.3	2.74, 6.59	< 0.001
**Dams and irrigated rice fields**					
Agro pastoralists	111 (55.7)	90 (44.3)	1.00		
Nomadic pastoralists	40 (18.9)	162 (81.1)	5.0	3.21, 7.79	< 0.001
**Presence of dambos**					
Agro pastoralists	142 (73.4)	59 (26.6)	1.00		
Nomadic pastoralists	45 (24.0)	157 (76.0)	8.4	5.36, 13.16	< 0.001
**Bushy vegetation**					
Agro pastoralists	133 (66.2)	68 (33.8)	1.00		
Nomadic pastoralists	49 (24.3)	153 (75.5)	6.1	3.96, 9.43	< 0.001
**Presence of rivers and streams**					
Agro pastoralists	141 (70.1)	60 (29.1)	1.00		
Nomadic pastoralists	36 (17.8)	166 (82.2)	10.8	6.77, 17.34	< 0.001
**Animal movement**					
Agro pastoralists	102 (50.2)	99 (49.3)	1.00		
Nomadic pastoralists	65 (28.9)	137 (71.1)	2.2	1.45, 3.25	0.001
**Seasons**					
Agro pastoralists	101 (50.7)	100 (49.7)	1.00		
Nomadic pastoralists	61 (30.2)	141 (69.8)	2.3	1.55, 3.51	0.001

*OR* Odds ratio, *CI* Confidence interval; Statistically significant at *p* < 0.05

## Discussion

No documented evidence of RVF outbreak has been reported in Nigeria. However, this study found evidence of silent RVFV circulation in all breeds, ages, and sex of pastoral cattle, with overall animal serological RVFV IgM prevalence of 5.6%. This could indicate recent natural exposure to the virus. Thus, absence of clinical signs in animals cannot exclude silent circulation of the virus in them and can be interpreted as possible recent infections in them. Previous studies have shown that anti-RVFV IgM antibodies persist until 45 days after infection in 50% of animals [2]. The silent circulation of active RVFV has previously been reported in slaughtered ruminants in Nigeria [28]. The observed higher prevalence in nomadic pastoral cattle (7.4%) than in agro-pastoral animals (3.8%) could be due to long-distance movements and exposures during grazing and watering, which are characteristic of nomadic cattle herds. Animal movements during grazing have been reported to be risk factor for RVF occurrence and spread in West Africa [29].

We found no significant influence of intrinsic factors (breed, age, sex, and production system) on RVF occurrence in the cattle herds. Contrary, previous studies reported natural correlation between RVFV seropositivity and age of animals [30, 31]. However, our findings are consistent with results of some studies that also reported no significant difference on seropositivity between male and female animals [32, 33]. No statistical significance observed could be due to the small sample size of the sampled animals in this study, derived from some limitations.

The use of indigenous knowledge of local communities is a viable undertaking in epidemiology because of its potential to support disease surveillance, early warning systems, and preventive measures thereby substantially mitigating risks of infectious diseases [34]. This study found common sources of information about RVF to be relations of the pastoralists and radio. Radio programmes are crucial in the dissemination of epidemiological information on diseases among pastoralists, who largely depend on radio to get information about livestock diseases. The use of radio as an efficient media for dissemination of information to educate livestock keepers on RVF has been substantiated [27, 35, 36].

Although respondents have heard about RVF, low positive responses on the disease epidemiology were observed, indicating that there were low levels of knowledge about it. Except for anorexia in animals, high mortality in newborns, and sudden onset of abortions in cows that had high positive responses, other clinical manifestations and symptoms of the disease in animals were characterized by low proportions of knowledge level responses. Also, there were few pastoralists with positive knowledge about modes of transmission of RVFV in animals, except for bites of infected mosquitoes that had high positive responses. These findings are consistent with results of a study that reported low knowledge among livestock keepers in Sudan, about vectors spreading RVF, signs and symptoms in animals [27]. Studies have also reported pastoralists in Kenya and Tanzania having limited knowledge about the symptoms and modes of RVFV transmission [37]. Low knowledge levels observed in this study could be attributed to absence of educational programmes targeted at livestock farmers on emerging zoonotic diseases in Nigeria.

This study found 62.8% of participants without formal education. Possession of formal education is very important as it creates opportunities for exchange of ideas among farmers on livestock diseases through seminars and workshops. Low knowledge about RVF could be also attributable to low formal education levels among pastoralists, which can predispose to low understanding about its zoonotic nature [38].

There was a significant influence of seasons and socio-ecological factors on RVF occurrence in pastoral cattle herds. Indeed, ecological factors of climate and landscape features can predispose to mosquito availability and population dynamics, which can consequently influence the emergence of RVF [39, 40]. Significant risk factors identified include: high mosquito availability, high cattle concentration, high rainfall, presence of ‘dambos’ and irrigated rice fields, availability of bushy vegetations, presence of rivers and streams, animal movement, and seasonal variables. High cattle concentration has been previously reported as risk factor for RVF transmission [41]. Ecological factors of climate, water bodies and other landscape features (such as forest, shrub, and agricultural areas) influence availability and population dynamics of vectors of RVF [42, 43]. The presence of temporary water bodies and floodplains, and forested or shrubby areas, artificial water bodies (such as dam and irrigated rice fields) are known to be predisposing factors for RVF occurrence in western and eastern Africa [42, 44, 45].

The results of this study have shown that pastoralists possessed low knowledge about RVF as a zoonotic disease. Educating pastoralists on the disease’s public health impacts that include mild illness with fever, headache, and myalgia, as well as severe cases of either retinitis with permanent vision loss or haemorrhagic forms that may lead to death [46, 47] is needed. Interventions that will enable pastoralists live in separate locations from animals are also required. Acting to address challenges caused by RVF in humans is essential because increase in seropositivity of the virus remains uncertain due to absence of routine surveillance data in Nigeria. On the basis of available estimates and likely geographical distribution associated with the risk factors, the number of animals with RVFV may largely exceeds the number affected by other zoonotic health challenges, such as brucellosis, bovine tuberculosis, antimicrobial residues, and resistance, that have received greater attention, funding, and resources.

Although there was RVFV specific IgM seroprevalence, major limitation was the relatively small sample size of animals, which might have undermined significant effects of independent variables on outcome variables during the univariable analysis of intrinsic determinants. A longitudinal cohort study involving large number of animals is advocated to clarify the epidemiology of the disease, with particular consideration for correlation of seroprevalence burden with intrinsic factors. The lack of full adjustments for pastoral cattle herds clustering in the designed random sampling was a limitation. However, the use of central tendency measures is valuable enough to tolerate the imperfections in the confidence intervals. Furthermore, questionnaire was also used for data collection, but pre-tested prior to actual data collection, to improve accuracy, quality control and ensured that no information was lost in the process.

## Conclusions

The results of this study illustrate recent circulation of RVFV in pastoral herds of Nigeria and needs urgent interventions. This study highlighted low levels of knowledge about RVF among surveyed pastoralists. The challenging gaps, including influence of socio-ecological risk factors, call for health education of these vulnerable populations about the socio-economic and health threats of RVF in the pastoral herds. For better understanding of RVF epidemiology, further investigations on the vector dynamics and livestock movements within Nigeria and across its borders are needed. To achieve food security and public health, the identified influencing risk factors will require cross-disciplinary collaborations for surveillance and control of the disease.

## Methods

### Ecological setting of study area

**Fig. 2:**
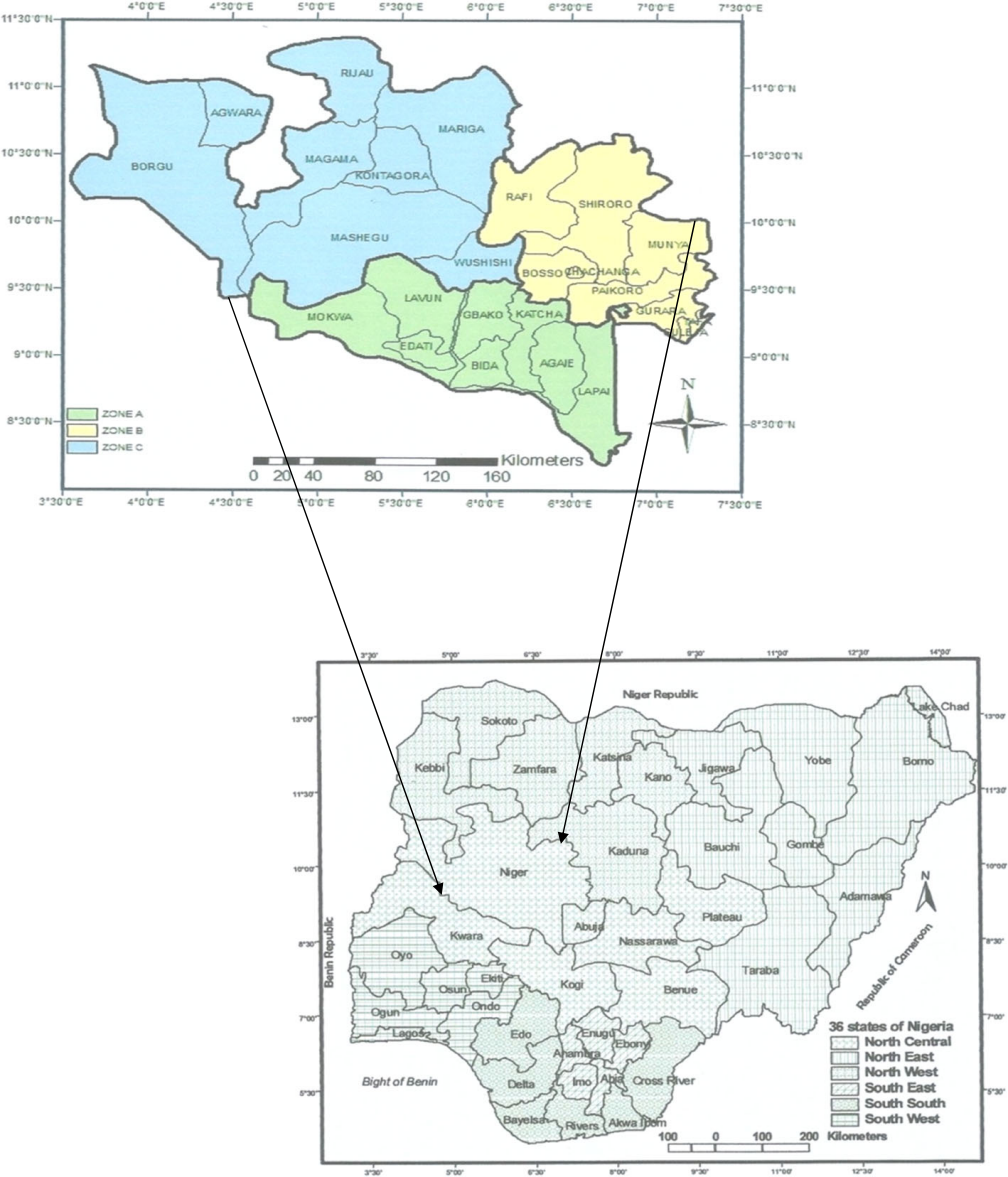
Map of Niger State and its location at the North-central zone of Nigeria. Source: Alhaji et al., *PLOS Negl Trop Dis* 2018a; 12(10):e0006858 [48]. It is not under copyright

The study was conducted in Niger State in the Southern Guinea Savannah zone of Nigeria, between latitudes 8° 20′ N and 11° 30′ N, and longitudes 3° 30′ E and 7° 20′ E. The state serves as transit routes for pastoral herds on seasonal transhumance movements between northern and southern parts of Nigeria. It has three designated Agro-ecological zones: southern, eastern and northern zones, with variable climatic conditions (Fig. 2). These zones are characterized by many rivers, streams and ponds, fadamas for rice farming and four hydroelectric dams. There are also Kainji National Game Reserve and many transnational stock routes.

The state experiences two distinct seasons: rainy season (April to October) and dry season (November to March), with mean annual rainfall of about 150 cm spanning for a period of approximately 180 days. It has average annual temperature range of 22 °C to 39 °C and relative humidity of about 58.6%. These ecological variables predispose the state to annual flooding and consequently provide suitable breeding environments for vectors of vector-borne diseases, such as RVF, in water-filled topographic depressions, the ‘dambos’. The state has an estimated cattle population of 2.5 million, which are mostly in the custody of pastoralists [49].

### Study design and target population

A cross-sectional study was conducted in pastoral herds, herding local breeds of cattle in two production systems in North-central Nigeria, between October 2017 and September 2018. Both serological and questionnaires tools were used for sample collection. For seropositivity investigation, average nomadic and agro pastoral herd sizes were 50 and 25 cattle, respectively. Sampled cattle were of both sexes, aged at least 1 year to exclude the effect of colostral antibodies and no vaccination history. Accessibility of herds was also considered, with insecure areas being excluded. Using questionnaire tool, age eligibility for pastoral household heads that participated was 20 years or above. They were expected at these ages to possess existing veterinary knowledge on livestock health management and diseases risk factors [50].

For the purpose of this research, a nomadic production system was defined as management that kept mainly cattle and took part in year-round movements of herds over large ranges for grazing without a permanent homestead. An agro-pastoral production system was a semi-settled herd with small number of cattle, cultivating few crops and having limited movements on low range grazing within their environments.

### Sample size and sampling procedure

For animal seroprevalence, sample size was determined using random sampling for finite population, with power set at 11.3% [48], 6% desired absolute precision at 95% confidence level. A sample size of 107 cattle was obtained. Sample size of households for questionnaire administration was determined using the same approach, with power set at 50% frequency of response; margin of error was 5% at 95% confidence level. A sample size of 384 households was obtained. To take care of non-response, a 5% contingency was added. Thus, 403 household heads were targeted for data collection.

A multi-stage sampling method was carried out. For questionnaire administration, three agro-ecological zones were purposively considered in the first stage. In the second stage, 15 settlements were selected for each production system (30 pastoral settlements in all) across the State, with five from either nomadic or agro pastoral herds in each Agro-zone. In the final stage, 134 pastoral households (67 from either group) were randomly selected in each zone. A total sample of 403 respondents, made up of 202 nomadic and 201 agro-pastoralists, were selected. For the seropositivity, 10 herds (5 nomadic and 5 agro-pastorals) were purposively selected in each zone. Also, a minimum of 3 cattle were randomly selected proportionately to the size of each herd. Agro-ecological zones A and C: 35 cattle each; and zone B: 37 cattle.

### Data collection: serum samples and serological analysis

Blood samples were collected from 107 cattle in the three agro-ecological zones of Niger State. During sampling, ages of the animals were recorded, and if the herder was not aware of an animal’s age, the dentition of the cattle was used to estimate the age. Sampled cattle were classified according to age: 1–3 years and > 3 years. Blood samples (5mls) were collected in dry vacutainer tubes from the jugular vein of each animal. Each vacutainer tube was labeled and individual animal information recorded. The collected blood samples were kept at 4 °C for 12 h to allow blood clot, centrifuged at 3000 g for 10 min for erythrocytes sedimentation and serum formation. The sera were transferred into new vials and labeled before being stored at -20 °C until further processing.

Serological analysis using IgM capture ELISA was conducted. To detect recent infection (IgM), all samples were tested using ID Screen RVF IgM ELISA (ID-Vet Innovative Diagnostics, Grabels, France) according to the manufacturer’s instructions. The test was considered valid when the mean value of the positive control OD (ODPC) was greater than 0.35 and the ratio of mean values of the positive and negative control ODs (ODPC and ODNC) was greater than 3. The sample was considered positive when the competition percentage was greater than or equal to 50%, doubtful when between 40 and 50%, and negative when ≤40%. All doubtful samples were considered as negative in this study.

### Data collection: questionnaire administration

We developed a structured questionnaire with mostly categorical questions to ease data processing and improve precision of responses. It was interviewer-administered by eight trained animal health technicians and supervised by the authors. The questionnaire consisted of four sections: demographic characteristics of respondents (6 questions); herd biodata (4 questions); existing knowledge about RVF (9 questions); and socio-ecological predisposing factors of RVFV occurrence in cattle herds (9 questions). The questionnaire was originally designed in English and verbally translated to local *Hausa* language during administration for respondents without formal education (Suppl. 1).

Questionnaire was pre-tested on 15 pastoral cattle herds’ settlements before final administration, and identified problems were eliminated and final high quality data collected. To achieve maximum response, advocacy visit was made to the leader (*Dikkos*) of each pastoral settlement a week prior to data collection and permission obtained. Respondents were assured of voluntary participation, confidentiality of responses and the opportunity to withdraw at any time without prejudice in line with the World Medical Association Declaration of Helsinki [51]. Informed consent was obtained either by signatures (for literates) or thumb-printings (for illiterates) on a sheet before questionnaire administration and none declined to participate.

### Data management and statistical analysis

Data from the field and laboratory were summarized into Microsoft Excel 7 (Microsoft Corporation, Redmond, WA, USA) spreadsheets and stored. Descriptive and analytical statistics were used. Frequencies and proportions were used for descriptive analysis. Categorical variables were presented as proportions and their associations determined by bivariate analysis using Chi-square tests. Associations were analyzed by univariable tests and multivariable logistic regressions analysis.

RVFV seropositivity in animals was measured as the proportion of animals presenting antibodies against RVFV to the total number of animals in the target population. To assess associations, demographic characteristics of animals and socio-ecological factors were the independent (explanatory) variables. Identified seropositivity and seronegativity as well as pastoralists’ categorical responses to questions in questionnaire formed the dependent (outcome) variables. All explanatory and outcome variables were initially screened by univariable analysis using Chi-square tests [52] or Fisher’s exact test, where appropriate. Likelihood stepwise backward multivariable logistic regressions model was built by adding variables in a backward selection process in order to start with those with significant p-value from the univariable analysis. This was used to control for confounding and test for effect modification. Variables with a p-value more than 0.05 on the univariable analysis were not included in the final model. The EpiInfo 3.4.3 (CDC, Atlanta, GA, USA) and OpenEpi version 2.3.1 [53] statistical packages were used for statistical analyses. A *p* < 0.05 was considered statistically significant in all analyses.

## Supplementary information

**Additional file 1.**

## Data Availability

The data analyzed during this study are available from the corresponding author on reasonable request.

## Abbreviations

RVF: Rift Valley Fever
RVFV: Rift Valley fever virus
ELISA: Enzyme-linked immunosorbent assay
OD: Optical density
IgM: Immunoglobulin M
CI: Confidence interval
*X*^*2*^: Chi-square
OR: Odds ratio
