# Supplementary material for: Seropositivity and associated intrinsic and extrinsic factors for Rift Valley fever virus occurrence in pastoral herds of Nigeria: a cross sectional survey
Source: BMC Vet Res. 2020 Jul 14;16:243. doi: 10.1186/s12917-020-02455-8 (PMC7359261; doi:10.1186/s12917-020-02455-8)
Supplement: Supplementary file 1 — Additional file 1. [file 12917_2020_2455_MOESM1_ESM.docx]

**Supplementary File 1** Questionnaire

This questionnaire is meant to assess pastoralists’ existing knowledge about Rift Valley fever (*Gabi-gabi*) in pastoral cattle herds of Niger State, North-central Nigeria and you have been identified to participate. Participation is optional and all information given will be kept strictly confidential. *PLEASE* ***tick one or more*** *appropriately and* ***write*** *where necessary****.***

Name of interviewer__________________________ Date of interview_______________

1. **Demographic Information**

A1. Name of interviewee: ____________________________

A2. Age (in years): _____________

A3. Gender: a. Male ( ) b. Female ( )

A4. Marital status: a. Married ( ) b. Single ( ) c. Widow ( )

A5. Occupation: a. Nomadic pastoralist ( ) b. Agro-pastoralist ( )

A6. Highest formal education:

1. None ( ) b. Primary ( ) c. Secondary ( ) d. Tertiary ( )
2. **Herd Information**

B1. What is the total number of cattle in your herd (herd size)? …………………….

B2. What type of cattle breeds do you rear in your herd? a. Local breeds ( ) b. Exotic breeds ( ) c. Mixed breeds ( ) d. All of the above ( )

B3. What species of cattle do you herd? …………………………………………………………..

B4. What type of husbandry management system do you practice for your herd?

1. Semi-extensive system (short distance grazing with feed supplementation) ( )
2. Extensive system (long distance grazing without feed supplementation) ( )
3. **Existing Knowledge about Rift Valley fever**

C1. Have you ever heard about Rift Valley fever (*Gabi-gabi*)? No ( ) Yes ( )

C2. If Yes, from what source?

1. Radio ( ) b. Friends ( ) c. Relations ( ) d. Community meetings ( ) e. Veterinary/health authorities ( ) e. Others (specify)­­­­­­­­­­­­­­­­­­­­­­­___________________

C3. Can Rift Valley fever affect cattle? No ( ) Yes ( )

C4. If Yes, what are the clinical signs observed in the herd? a. Anorexia ( ) b. High fever ( ) c. Listlessness in newborns ( ) d. Collapse and sudden deaths of newborns ( ) e. Abortions in pregnant animals ( ) f. Profuse fetid diarrhoea ( ) g. Mucopurulent nasal discharge ( )

C5. Is Rift Valley fever transmissible from animals to humans (zoonosis)? No ( ) Yes ( )

C6. If Yes, what are the clinical symptoms manifested in humans?

1. High fever ( ) b. Headache ( ) c. Muscular pains ( ) d. Blurred vision ( ) e. Backache ( ) f. Internal bleeding ( ) g. All of the above ( )

C7. Is Rift Valley fever routinely found (endemic) in this community? No ( ) Yes ( )

C8. Can Rift Valley fever be transmitted by mosquitoes to animals? No ( ) Yes ( )

C9. Can RVF be transmitted by bites of other flies (tsetse, stable flies, ticks, etc) to animals? No ( ) Yes ( )

1. **Socio-ecological Factors predisposing to RVF virus occurrence in Cattle Herds**

Based on your existing knowledge, what is/are the risk factors that predispose to occurrence of RVF in your herds?

D1. Availability of mosquitoes: No ( ) Yes ( )

D2. High cattle concentration : No ( ) Yes ( )

D3. High rainfall: No ( ) Yes ( )

D4. Dams and irrigate rice fields: No ( ) Yes ( )

D5. Presence ‘of dambos’: No ( ) Yes ( )

D6. Bushy vegetation: No ( ) Yes ( )

D7. Presence of rivers and streams (floodplains): No ( ) Yes ( )

D8. Animal movement: No ( ) Yes ( )

D9. Seasons: No ( ) Yes ( )

Thank you for the kind responses

**For further information, please call +234(0)803 595 0915**
